# Secretome and Pathway Analysis of Stress Induced Disc Degeneration In Vitro

**DOI:** 10.1002/jsp2.70165

**Published:** 2026-03-27

**Authors:** Exarchos Kanelis, Andrea Nüesch, Joseph Snuggs, Christine Lyn Le Maitre, Leonidas G. Alexopoulos

**Affiliations:** ^1^ Protavio, Demokritos Technology Park Agia Paraskevi Greece; ^2^ Biomedical Systems Laboratory, School of Mechanical Engineering National Technical University of Athens Athens Greece; ^3^ Division of Clinical Medicine, School of Medicine and Population Health University of Sheffield Sheffield UK

## Abstract

**Background:**

Intervertebral disc degeneration (IDD) involves complex inflammatory and oxidative stress pathways within nucleus pulposus (NP) cells, leading to catabolic remodeling and loss of disc function. Traditional single‐analyte assays capture only a small part of these coordinated networks. Leveraging the multiplex capability of Luminex, this study uses targeted immunoassays to simultaneously quantify 73 extracellular proteins and 21 intracellular signaling molecules in primary human NP cells stimulated with IL‐1β, Pam2CSK4, or hydrogen peroxide, enabling comparison of shared and stimulus‐specific responses.

**Methods:**

Human NP cells isolated from patients undergoing spine surgery were plated in monolayer or encapsulated in alginate and stimulated for 1 week with either: 100 pg/mL or 1 ng/mL Interleukin (IL)‐1β, or the Toll‐Like Receptor (TLR) 2/6 agonist Pam2CSK4 at 100 ng/mL, or 50 μM Hydrogen Peroxide (H_2_O_2_). The role of the stimuli was investigated using mRNA expression analysis and Luminex multiplex immunoassays.

**Results:**

TLR2/6 agonist and IL‐1β stimulation significantly up‐regulated IL‐6, IL‐8, IL‐1β, and MMP‐3 mRNA expression, while H_2_O_2_ failed to induce any changes. The secretome showed that IL‐8, IL‐6, MMP‐7, Intercellular Adhesion Molecule 1 (ICAM‐1), and Growth‐Regulated Oncogene‐α (GRO‐α) were the most responsive proteins across treatments and culture conditions. Intracellularly, phosphorylation of Jun Proto‐Oncogene AP‐1 Transcription Factor Subunit (cJUN), Nuclear Factor Kappa B (NF‐kB), and Inhibitor of Nuclear Factor Kappa B Alpha (IKBA) was observed following IL‐1β and Pam2CSK4 stimulation.

**Conclusions:**

Across inflammatory and innate immune activation and under both monolayer and 3D culture, a concise five‐marker panel, namely IL‐6, IL‐8, GRO‐α (CXCL‐1), ICAM‐1, and MMP‐7 captured NP cell responses with high consistency, while phosphoproteomics demonstrated convergence on NF‐κB/AP‐1 signaling. These markers are well established within disc‐degeneration pathways (inflammation, innate immunity, and ECM remodeling); thus, they offer practical, reusable readouts for comparative pharmacology, assay qualification, and early biomarker development in translational spine research.

## Graphical abstract

Integrated secretome and phosphoprotein profiling of human nucleus pulposus cells revealed that IL‐1β and TLR2/6 activation induce a shared degenerative program, defined by consistent upregulation of IL‐6, IL‐8, GRO‐α, ICAM‐1, and MMP‐7 and convergence on NF‐κB/AP‐1 signaling.

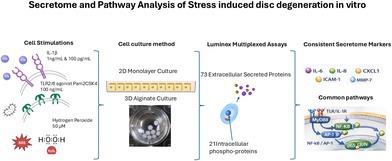

## 1 Introduction

Low back pain is one of the leading causes of disability worldwide, with projections indicating that its prevalence will continue to rise [1, 2]. It is estimated that up to 80% of individuals in Western countries will experience back pain at some point in their lives, making the condition highly impactful in socio‐economic terms due to increased healthcare costs and loss of productivity [1]. Low back pain is strongly associated with intervertebral disc (IVD) degeneration (IDD), a prevalent condition marked by the progressive structural and functional decline of IVDs [3]. IVDs are crucial structures within the human spine, withstanding daily loading and providing flexibility of the spine, positioned between vertebrae. Each IVD consists of a gelatinous NP surrounded by a fibrous annulus fibrosus (AF) and cartilagenous endplates (CEP) superiorly and inferiorly. Together, these structures maintain spinal stability and facilitate movement [4]. However, IDD results in reduced disc height, decreased hydration, and compromised structural integrity, frequently leading to chronic low back pain and diminished quality of life [5].

Despite its prevalence, the mechanisms underlying IDD, while extensively studied, remain the focus of active research due to the complex interplay of biological, mechanical, and biochemical factors. This complexity underscores the need for further research to elucidate the molecular mechanisms involved in IDD progression. Of particular interest are the roles of inflammatory mediators, such as interleukin (IL)‐1β, tumor necrosis factor (TNF), and IL‐6, alongside innate immune responses and oxidative stress (OS), as these factors have been highlighted as critical contributors to the degenerative process [6]. NP cells, which normally reside in an immune‐privileged environment, can secrete cytokines and chemokines themselves, further exacerbating local degenerative processes [7].

Among these mediators, interleukin‐1β (IL‐1β) is recognized as a key driver of catabolic changes in NP cells. IL‐1β upregulates matrix‐degrading enzymes such as MMPs and ADAMTS, while suppressing the synthesis of essential extracellular matrix (ECM) components, leading to structural breakdown of the disc [8]. Even at concentrations as low as 100 pg/mL, IL‐1β has been shown to significantly alter NP cell secretome, increasing the production of multiple cytokines and chemokines, including IL‐6, CCL2, and CXCL8 [9]. Recent studies which have quantified the levels of IL‐1β produced from native NP tissue have shown that 100 pg/mL represents a physiologically relevant concentration within human degenerate NP tissues [10].

Components of the innate immune system also play an important role in disc degeneration. Toll‐like receptor 2 (TLR2), particularly in association with its co‐receptor TLR6, recognizes both pathogen‐associated molecular patterns (PAMPs) and damage‐associated molecular patterns (DAMPs), initiating catabolic cascades in NP cells [11, 12]. Relevant PAMPs for TLR2/6 include bacterial lipoproteins and lipopeptides from gram‐positive bacteria such as *Cutibacterium acnes* (*C. acnes*), which has been detected in degenerated discs [13] and shown to stimulate NP cells to secrete IL‐6, TNF‐α, CCL2 (MCP‐1), and CXCL8 (IL‐8), as well as upregulate matrix‐degrading enzymes including MMP‐1, MMP‐3, and MMP‐13 via the TLR2–NF‐κB and TLR2–JNK pathways [12, 14, 15]. Relevant DAMPs include ECM degradation products such as soluble biglycan, which acts as an endogenous danger signal activating TLR2 (and TLR4) and the NLRP3 inflammasome, further amplifying cytokine release and catabolic enzyme production [16]. These responses contribute to proteoglycan loss, ECM breakdown, and chronic inflammatory signaling in IVD tissue.

Another critical factor implicated in IDD pathogenesis is OS [17]. OS in IVDs is evidenced by the buildup of advanced glycation end‐products, such as carboxymethyl‐lysine and pentoside, resulting from the oxidative modification of glycosylated proteins [18, 19]. Hydrogen peroxide has been detected in human NP tissue at concentrations of approximately 0.25 μmol/20 mg tissue, indicating that NP cells are physiologically exposed to this type of reactive oxygen species (ROS) [18, 19]. Hydrogen peroxide is commonly used in vitro to simulate OS in NP cells, allowing researchers to investigate its effects on cell viability, ECM synthesis, and activation of stress‐related signaling pathways.

Based on these observations, this study employed IL‐1β to model inflammatory cytokine signaling, the synthetic TLR2/6 agonist Pam2CSK4 to mimic PAMP/DAMP‐mediated innate immune activation, and H_2_O_2_ to simulate OS. Together, these stimuli capture key pathological mechanisms relevant to human NP cell degeneration and senescence.

While previous studies have examined the effects of IL‐1β, TLR2/6 activation, or OS on NP cells, most have focused on a limited subset of extracellular inflammatory mediators or matrix‐degrading enzymes. There is a lack of integrated analysis combining both extracellular signaling proteins and intracellular signaling pathway molecules under these key degenerative stimuli. Such combined profiling is essential to capture the interplay between upstream receptor‐mediated responses and downstream signaling cascades, which together drive catabolic and pro‐inflammatory changes in IDD. Furthermore, prior studies are often completed in monolayer or within 3D cultures but under high glucose and high oxygen conditions [2, 7, 20]. Thus, there is a lack of studies which determine whether the responses seen in monolayer are representative of those within a more physiological environment where NP cells have been re‐differentiated, namely within 3D alginate cultures, under physioxia and low glucose conditions. We hypothesize that IL‐1β, TLR2/6 activation, and OS each drive distinct yet partially overlapping extracellular and intracellular molecular profiles in NP cells, and that integrated analysis will identify key pathways and mediators contributing to the inflammatory and catabolic environment characteristic of IDD.

## 2 Materials and Methods

### 2.1 Study Rational of Experimental Design

This study investigates the influence of potential IDD regulators within human NP cells in vitro providing an integrated analysis of secretomics and intracellular signaling pathways. Adopting two field‐standard paradigms aligned with the ORS Spine multi‐lab harmonization consensus: 2D monolayer expansion in high‐glucose media at ~21% O_2_, and 3D alginate re‐differentiation in low‐glucose DMEM at 5% O_2_ (physioxia), using the consensus 3D medium and O_2_ settings to better approximate the in vivo niche [21]. This design combines responses between a dedifferentiated 2D state and a more physiological, re‐differentiated 3D context within physiological glucose and O_2_ conditions.

Stimuli and doses were selected based on prior work, namely IL‐1β was utilized at two doses: 100 pg/mL and 1 ng/mL. Where100 pg/mL was selected based on prior human NP studies demonstrating robust cytokine/chemokine induction at this concentration [7], and native tissue production at these levels [10], while 1 ng/mL served as a higher reference dose and mimicked more commonly utilized doses utilized [9, 11]; Pam2CSK4 (100 ng/mL) was chosen to produce reliable TLR2/6 activation; and H_2_O_2_ (50 μM) was selected following initial viability testing to determine a concentration where by OS could be modeled without significant viability loss. Collectively, these doses balance physiological relevance (low‐dose IL‐1β) with signal detectability (higher IL‐1β, TLR2/6 agonism) and non‐lethal OS (50 μM H_2_O_2_), enabling us to compare overlapping versus stimulus‐specific pathways across contexts.

Following stimulation, the outcome measures focused primarily on the use of multiplex Luminex‐based targeted immunoassays to simultaneously quantify 73 extracellular proteins in the secretome and 21 intracellular signaling molecules. This approach enabled comparison of both shared and stimulus‐specific responses, as well as the identification of potentially conserved protein‐marker panels in the secretome and phospho‐activation profiles. These endpoints may be valuable for comparative pharmacology, potency‐assay development, and early biomarker discovery in disc‐degeneration research, while also supporting reproducible comparisons across studies.

### 2.2 NP Cell Extraction and Expansion

Human IVD tissue was obtained from patients undergoing surgery with informed consent during spinal surgery at Northern General Hospital or Claremont Hospital in Sheffield (UK). Local ethics approval was given for this work by Sheffield Research Ethics Committee (09/H1308/70) (IRAS approval: 10226). Surgical samples from 7 different individuals were used in this study (Table S1). A portion of the NP tissue was set aside for histopathological grading prior to enzymatic digestion. This tissue was fixed in 10% neutral‐buffered formalin for 24 h, dehydrated through graded ethanol, embedded in paraffin, and sectioned at 5 μm thickness. Sections were stained with hematoxylin and eosin (H&E), Alcian Blue, and Safranin O–fast green. Degeneration grade was assessed using the standardized histopathology scoring system for human IVD degeneration described by Le Maitre et al., which evaluates morphological features, matrix organization, proteoglycan content, and cellularity [22]. Cell isolation was carried out following the standardized protocol described previously [21]. Briefly, after tissue sample acquisition and prior to digestion, the tissue was washed twice with PBS and was morphologically separated into NP and AF tissue. NP tissue was weighed prior to digestion to enable calculation of cell yield per g of tissue. Human NP cells were isolated using collagenase type II (Sigma Aldrich, Gillingham, UK, 17101‐015) at a concentration of 64 U/mL (0.5 mg/mL) in serum‐free hg‐DMEM containing 1% P/S at 20 mL/g tissue for 4 h on a 600 rpm orbital shaker at 37°C [21]. Following digestion, NP tissue was removed using a 70 μm cell strainer and the solution was centrifuged at 400 g for 10 min, and the supernatant was discarded. The cell pellet was then resuspended in complete medium containing hg‐DMEM (HG‐DMEM, Invitrogen: 10569010 HG) supplemented with 10% v/v heat‐inactivated fetal calf serum (FCS, LifeTechnologies, Paisley, UK), 1% v/v P/S (P/S, Gibco, 15070‐063), 1% v/v L‐Glutamine (Invitrogen: 25030‐024), 2.5 μg/mL Amphotericin B (Sigma, A2942), 25 μg/mL L‐ascorbic acid 2‐Phosphate (AA, Sigma, A5960). Cell viability and counting were determined via NucleoCounter (NC2000, ChemoMetec) and cells were plated at a concentration of approximately 10 000 cells/cm^2^. The confluence of the flasks was monitored daily and at around 85% confluency, the cells were split 1 to 3. The cells were maintained in monolayer until passage 3 at 37°C in a humidified atmosphere containing 21% O_2_, 5% CO_2_, with media renewal 3 times a week [11].

### 2.3 Evaluation of H_2_O_2_ Effect on NP Cell Viability

The effect of different concentrations of H_2_O_2_ on the viability of human NP cells was investigated using the luminescence‐based cell viability assay CellTiter‐Glo (Promega). Briefly, 10 000 cells were plated in 5 replicates in a 96‐well plate and were left to adhere overnight at a final volume of 100 μL expansion media. The concentrations of H_2_O_2_ assayed were 0, 15.7, 31.25, 62.5, 125, 250, 500, 1000 μM. CellTiter‐Glo was used as recommended by the manufacturer and read on a Varioskan absorbance plate reader (Varioskan LUX Multimode Microplate Reader, Thermo Scientific).

### 2.4 Monolayer Culture and Treatments

For monolayer stimulations, cells were plated in 6‐well plates at a concentration of 2 × 10^5^ cells per well and were stimulated with complete media as well as media supplemented with either 100 ng/mL Pam2CSK4 agonist, 100 pg/mL IL‐1β, 1 ng/mL IL‐1β or 50 μM Η_2_Ο_2_. The media and treatments were renewed on Day 1, Day 3, and Day 6 and samples were collected on Day 8. The Day‐8 readout integrates sustained responses under repeated stimulation (days 1, 3, 6). Monolayer expansion and stimulations were performed in high‐glucose DMEM at 21% O_2_, which reflects the commonly used conditions for NP cell expansion across laboratories [21].

### 2.5 Alginate Culture and Treatments

Since NP cells in monolayer culture are known to de‐differentiate, their responses may not fully represent the phenotypic response seen in vivo and hence further selected stimulations were repeated on NP cells which were firstly re‐differentiated in alginate bead cultures [21]. Cells at passage 3 were trypsinized, counted using a cell counter (NucleoCounter NC‐2000), and subsequently resuspended at a density of 4 × 10^6^ cells/mL in a sterile, filtered 1.2% (w/v) medium‐viscosity sodium alginate solution (Sigma A2033) prepared in 0.15 M NaCl as described previously [21]. Alginate cultures were then maintained in low glucose‐DMEM (1 g/L—Gibco 31600‐083) supplemented with 1% P/S, 2.5 μg/mL AmpB, 25 μg/mL L‐Ascorbic acid, 1% L‐Glutamine, 1% ITS‐X, 40 μg/mL L‐Proline, 1.25 mg/mL Albumax (complete 3D media) at 5% O_2_, 5% CO_2_, 37°C for 2 weeks, with a media change every 2 days to achieve phenotype recovery and mimic the in vivo micro environment before the initiation of stimulations. After the differentiation period of 2 weeks, the alginate beads were cultured with complete 3D media as control, or media supplemented with 100 ng/mL Pam2CSK4 agonist or 100 pg/mL IL‐1β for 1 full week. The media and treatments were changed at Day 1, Day 3, and Day 6 and the samples were collected at Day 8. The Day‐8 readout integrates sustained responses under repeated stimulation (days 1, 3, 6) and aligns with the time course studied in monolayer, enabling comparison across formats. Time‐course experiments were beyond the scope of this study.

### 2.6 RNA Extraction and Real‐Time PCR Analysis

After treatment of NP cells in monolayer was completed, the media was collected, cells were washed twice with PBS and RNA was extracted and further reversed transcribed to DNA as reported previously [21]. Finally, the cDNA was diluted 1:10 by adding 450 μL of water. A mastermix comprising 5 μL Taqman FAST universal mastermix, 2.5 μL sterile water and 0.5 μL Taqman gene expression assay was prepared for each target gene investigated (Table S2). From this mastermix, 8 μL was loaded onto wells of a 96‐well FAST PCR plates alongside 2 μL cDNA sample. Plates were run on a QuantStudio using 50 cycles of denaturation at 95°C for 1 s followed by annealing and extension at 60°C for 20 s. Measurements were performed in duplicate, and the average CT value was calculated. The relative gene expression level of each gene under investigation was determined using the 2^−ΔΔCT^ method by normalizing to the expression of two internal reference genes, *GAPDH* and *18S*, and then to the un‐stimulated control samples [23].

### 2.7 Luminex Assays—Secretome Analysis

For the Secretome analysis, 1 mL of conditioned media was recovered for each sample after exposure to the stress factors and was stored at −80°C. A multiplex assay comprising 73 analytes (Table S3), divided into six panels due to compatibility issues, was used to interrogate every sample. To determine the concentration of every soluble marker in the samples, an 8‐point standard curve was generated for each marker in the panels. Protein concentration in unknown samples was extrapolated based on a 5PL model generated by the Luminex software using the standards (Table S7). Assays with a value below the Limit of Detection (LOD) were replaced with LOD/sqrt(2) while missing values were replaced with the average of the replicates of the condition that were measurable. The assay was performed as described previously [24]. Briefly, the beads were first diluted to a 1× concentration using a protein‐free blocking buffer (Pierce Protein‐Free Blocking Buffer, #37573, Thermo Scientific), and 50 μL of beads (2500 beads per bead ID) were added to each well of a flat‐bottom plate. The plate was placed on a magnet for 1 min, the supernatant was removed, and the wells were washed with 100 μL of assay buffer (PR‐ASSB‐1x, Protavio, Greece). Prepared standards, blanks, and samples (35 μL each) were added, and the plate was incubated at room temperature for 1.5 h, shaking at 1000 rpm. After incubation, the wells were washed twice with 100 μL assay buffer, and 20 μL of detection antibodies were added and incubated for 1 h with shaking. Following this, the plate was washed twice, and 35 μL of diluted Streptavidin R‐Phycoerythrin Conjugate (#SAPE‐001, MOSS, USA) was added and incubated for 15 min before a final wash with 100 μL assay buffer. The microspheres were then resuspended in 130 μL of assay buffer and shaken for 5 min. Measurements were conducted using a Luminex FLEXMAP 3D platform (Luminex FLEXMAP 3D platform, Luminex a Diasorin company, Austin‐TX, USA), ensuring that at least 100 beads per analyte were measured to accurately estimate median fluorescence intensity (MFI). The media samples used for Secretome analysis were assayed without dilution or adjustment. The concentration of each marker was normalized to the total protein content of the corresponding well (BCA Protein Assay, BCA Protein Assay Kit—Thermo Fisher Scientific, Cat# 23225).

### 2.8 Luminex Assays—Phosphoproteomic Analysis

After the media was collected for secretome analysis the cells in monolayer were utilized for lysate generation. Cells were washed with cold PBS, and 200 μL lysis buffer (PR‐LYSB, Protavio, Greece) was added to the wells. The buffer was prepared according to Protavio's instructions by supplementing it with 1:100 protease inhibitors (Protavio, #PR‐PIC) and 1:50 PMSF (Sigma, #93482). Plates were shaken at 650 rpm for 20 min at 4°C, after which the lysates were collected in a 96‐well plate and stored at −80°C until final analysis. Prior to the phospho‐proteomic analysis, the total protein concentration in each lysate sample was determined using the Pierce BCA Protein Assay Kit (Thermo Fisher Scientific, Cat# 23225) following the manufacturer's protocol. Absorbance was measured at 562 nm using a Varioskan absorbance plate reader (Varioskan LUX Multimode Microplate Reader, Thermo Scientific). Before adjusting the concentration of cell lysates at 300 μg/mL using lysis buffer, the samples were centrifuged at 2700 g for 20 min to remove cell debris.

Phosphoproteomic analysis targeted 21 protein markers, split into two panels (Table S4). The phosphoproteomic analysis utilized a semiquantitative approach, presenting results as MFIs and reporting differences as a ratio of treatment versus untreated control (Table S8). To ensure assay validity, positive and negative control samples were matched on the plates, and a minimum ratio of 1.5 was required for the assay to be considered valid (Table S5). The assay steps and Luminex instrument were the same as in the Secretome analysis mentioned previously.

### 2.9 Statistical Analysis

Data analysis was conducted using R (version 4.3.1; R Core Team, 2022). The following R packages were used: tidyverse [25] for data cleaning, data manipulation and visualization and ggrepel [26] for improving layout in volcano plots. Biological replicate numbers for each assay were as follows: PCR, *N* = 6 donors; Luminex secretome analysis, *N* = 6 donors; Luminex cell signaling analysis, *N* = 5 donors; and secretome analysis in alginate, *N* = 3 donors. For each donor, cells were seeded in three independent wells (triplicates: *n* = 3) and analyzed as experimental replicates; no within‐well technical replicates were assayed. Given the limited number of biological replicates per assay, there were insufficient data points to reliably assess the normality of distributions using tests such as Shapiro–Wilk, which have low power at small *n*. Therefore, non‐parametric statistical methods were employed as a robust and conservative approach. When comparing more than two groups, a Kruskal–Wallis test followed by Dunn's multiple comparisons post hoc analysis and a Bonferroni multiple comparison correction was used to identify significant differences. For the statistical analysis, all gene expression, secretome, and phospho‐proteomic data were normalized to the average of the three control replicates of the same donor. Finally, for the secretome analysis, a fold change cutoff of ±1.5 combined with a *p* value threshold of 0.05 was applied for identifying differentially expressed proteins. It was assumed that a fold change of at least 1.5 times represents a potential biologically relevant alteration in protein expression. This threshold was selected to balance sensitivity and specificity (details for the donors in each treatment group can be found in Table S9).

## 3 Results

### 3.1 Effect of H_2_O_2_ on Human NP Cell Viability

At lower concentrations of H_2_O_2_ (approximately 50 μM), NP cell viability remained near 100%, indicating no significant decrease (Figure S1). A gradual decline in viability starting at 62.5 μM was observed; however, a significant decrease in viability was only observed at concentrations of 0.5 mM and over (*p* < 0.05) (Figure S1), with a significant decrease in viability following 0.5 mM to ~50% (*p* = 0.008, 95% CI: 16.26–77.61), and limited viable cells present following stimulation with 1 mM (*p* < 0.001, 95% CI:0–5.12) (Figure S1).

### 3.2 IL‐1β and Pam2CSK4 Promote Pro‐Inflammatory and Catabolic Gene Expression but Without Promoting Cellular Senescence

To explore the effects of the stimuli on NP cells, the expression profiles of six genes central to IVD inflammatory cytokine production, matrix degradation, and cellular senescence were examined. The selected genes included *IL‐1β*, *IL‐6*, *IL‐8*, *MMP‐3*, *p16* ^*INK4a*^, and *p21* ^*WAF1*^. As a preliminary assessment of overall treatment effects, a Kruskal–Wallis test was applied across the five experimental conditions for each gene. The omnibus test indicated significant differences between groups for *IL‐1β* (*p* < 0.001), *IL‐6 (p* < 0.001), *IL‐8* (*p* < 0.001), *MMP‐3* (*p* < 0.001), and *p16* ^*INK4A*^ (*p* = 0.022), whereas no significant difference was observed for *p21* ^*WAF1*^ (*p* = 0.13). These *p* values refer to the overall group effect and not to pairwise comparisons. Dunn's multiple comparison post hoc analysis was conducted to identify significant differences among groups.

**FIGURE 1:**
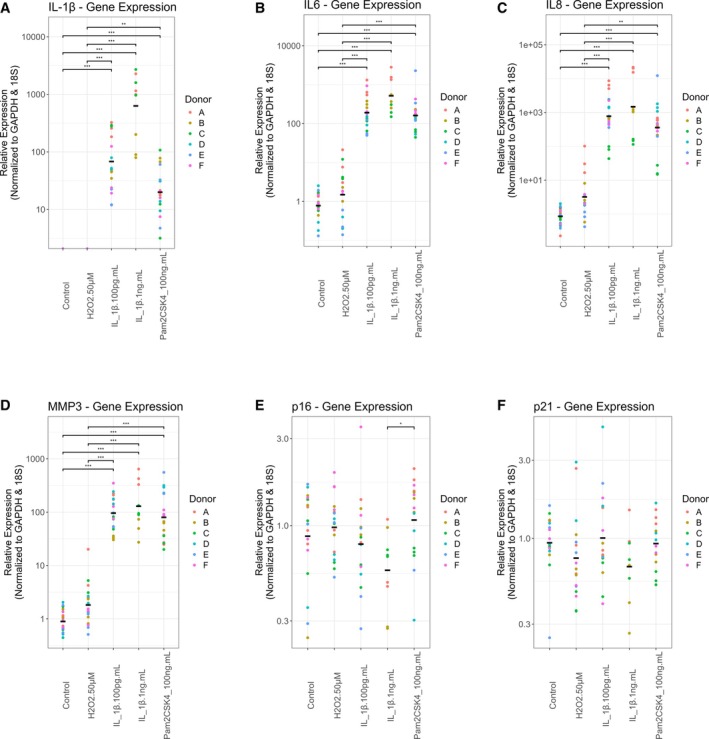
Relative, to untreated controls, expression of genes related to IDD, after stimulation of human NP cells in monolayer. (A) IL‐1β, (B) IL‐6, (C) IL‐8, (D) MMP‐3, (E) p16^INK4A^, (F) p21^WAF1^. The replicate samples of each patient donor are defined with the same color. The average expression for each treatment is shown (*N* = 3–6, *n* = 3). Kruskal–Wallis test and a Dunn's multiple comparisons post hoc test rank‐sum statistical test was performed with a *p* value < 0.05, which was considered statistically significant (*p* > 0.05: not significant, *p* < 0.05 and *p* > 0.01: *, *p* < 0.01 and *p* > 0.001: **, *p* < 0.001: ***).

Following stimulation of human NP cells with 1 ng/mL IL‐1β, mRNA expression of IL‐1β (*p* < 0.001), IL‐6 (*p* < 0.001), IL‐8 (*p* < 0.001), and MMP‐3 (*p* < 0.001) increased relative to untreated controls (Figure 1A–D). IL‐1β at 100 pg/mL produced a similar increase in these genes (IL‐1β *p* < 0.001; IL‐6 *p* < 0.001; IL‐8 *p* < 0.001; MMP‐3 *p* < 0.001) (Figure 1A–D). A comparable pattern was observed with 100 ng/mL Pam2CSK4, which also upregulated IL‐1β (*p* < 0.001), IL‐6 (*p* < 0.001), IL‐8 (*p* < 0.001), and MMP‐3 (*p* < 0.001) (Figure 1A–D). In contrast, p16^INK4a^ did not differ from control under any stimulation (*p* > 0.05; Figure 1E), although in Pam2CSK4 treated cells it was detected in higher levels compared to 1 ng/mL IL‐1β (*p* = 0.028). No differences were detected between Pam2CSK4 and IL‐1β for the four upregulated markers, indicating similar NP cell responses to these stimuli. Finally, H_2_O_2_ exposure did not significantly alter the expression of any gene examined (*p* > 0.05 for all; Figure 1A–F). p21^WAF1^ expression did not differ from the untreated control under any stimulation (*p* > 0.05; Figure 1F), and no post hoc pairwise tests were pursued.

### 3.3 IL‐1β at 100 pg/mL and 100 ng/mL Pam2CSK4 Induced a Similar Secretome Response in Monolayer Culture

The secretome of NP cells cultured in monolayer and treated with either 1 ng/mL IL‐1β, 100 pg/mL IL‐1β, 100 ng/mL Pam2CSK4, or 50 μM H_2_O_2_ was profiled using a panel of 73 proteins. Prior to performing differential secretion analysis, the baseline secretome levels of untreated human NP cells were investigated. This analysis aimed to capture variability among donors and evaluate potential responsiveness in the subsequent downstream analyses, where treated samples would be compared to these controls. Overall, anti‐inflammatory, ECM, angiogenic/neurotropic, regulator, pro‐inflammatory, chemokine, and “other” panels showed heterogeneous baselines across donors, with select markers (e.g., VEGF, PAI‐1, TIMP‐1, FST) consistently higher and others (e.g., M‐CSF) lower. Donor‐specific elevations (e.g., chemokines in donor C) are summarized in Figure S2.

**FIGURE 2:**
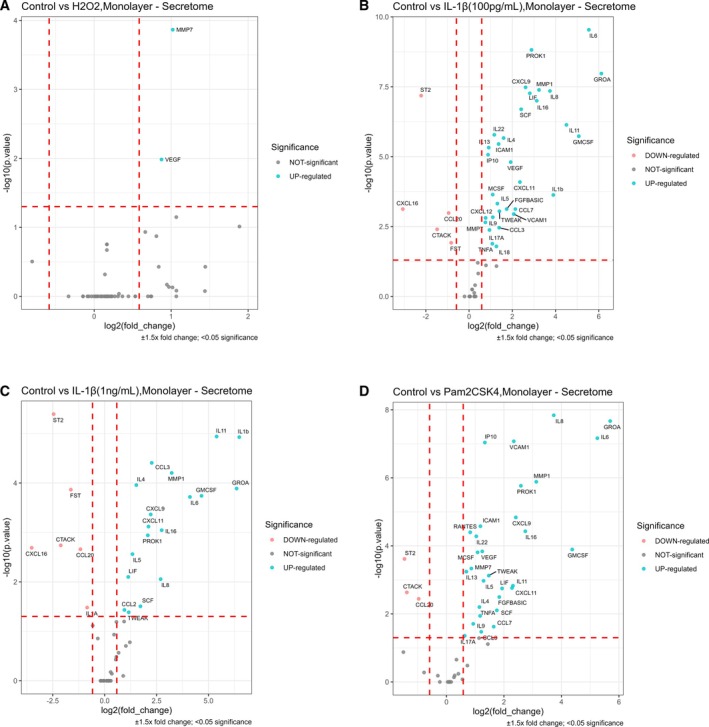
Differentially secreted markers after stimulating human NP cells with IL‐1β, Pam2CSK4 and H_2_O_2_ in monolayer. (A) 50 μM H_2_O_2_ versus control, (B) 100 pg/mL IL‐1β versus control, (C) 1 ng/mL IL‐1β versus control, and (D) 100 ng/mL Pam2CSK4 versus control. Group comparisons were conducted using Dunn's post hoc test, with a sample size of *N* = 3–6 donors and three technical replicates per stimulation. Protein expression levels were normalized to untreated controls, and the resulting plots depict the differences between treated samples and controls along with their adjusted *p* values. Markers were considered differentially expressed if their levels changed by at least ±1.5‐fold with a *p* value < 0.05. Additionally, protein levels were normalized to the total protein concentration for each well prior to analysis.

To determine secretome differences between treatment groups, a Kruskal–Wallis test identified 53 markers as significantly altered across the different treatments (*p* < 0.05) (Table S6), therefore these were subsequently selected for differential expression analysis. Treatment with H_2_O_2_ (50 μM) resulted in a significant increase for VEGF and MMP‐7 (Figure 2A).

Fifteen markers, namely CCL‐3, CXCL‐11, CXCL‐9, Granulocyte‐Macrophage Colony‐Stimulating Factor (GM‐CSF), GRO‐α, IL‐11, IL‐4, IL‐5, IL‐6, IL‐8, Leukemia Inhibitory Factor (LIF), MMP‐1, PROK1, Stem Cell Factor (SCF), TNF‐Related Weak Inducer of Apoptosis (TWEAK) were upregulated and three markers CCL‐20, Cutaneous T‐cell‐Attracting Chemokine (CTACK), Suppressor of Tumorigenicity 2 (ST2) were downregulated (Table 1) by both IL‐1β (at concentrations of 100 pg/mL and 1 μg/mL) and Pam2CSK4 (100 ng/mL) treatments (Figure 2B,D). Whilst 11 additional markers namely CCL‐7, b‐FGF, ICAM1, IL‐13, IL‐17A, IL‐22, IL‐9, IP‐10, Macrophage Colony‐Stimulating Factor (M‐CSF), Tumor Necrosis Factor‐α (TNFα), and VCAM1 were consistently upregulated following treatment with 100 pg/mL IL‐1β and Pam2CSK4 (Table 1). However, these markers were not elevated in response to 1 ng/mL IL‐1β or H_2_O_2_ treatment (Figure 2A,D). This selective upregulation underscores the distinct influence of lower physiologically relevant IL‐1β concentrations and Pam2CSK4 on inflammatory factor production and catabolic pathways in NP cells.

**TABLE 1:** Protein expression and statistical signif**ican**ce of NP cell secretions following IL‐1β, Pam2CSK4, and H_2_O_2_ Stimulation in monolayer cultures.

Monolayer		H_2_O_2_ vs. control		IL‐1β (100 pg/mL) vs. control		IL‐1β (1 ng/mL) vs. control		Pam2CSK4 vs. control	
Type	Protein. marker	Adj.*p*.value	Fold.change	Adj.*p*.value	Fold.change	Adj.*p*.value	Fold.change	Adj.*p*.value	Fold. change
Chemokine	CCL20	1.00E+00	1.12 ± 0.16	1.04E−03	0.52 ± 0.28	2.19E−03	0.45 ± 0.29	3.61E−03	0.51 ± 0.37
Chemokine	CCL3	1.00E+00	1.16 ± 0.67	3.47E−03	2.62 ± 1.67	3.92E−05	4.81 ± 2.21	3.36E−02	2.32 ± 1.54
Chemokine	CTACK	1.00E+00	1.24 ± 0.54	3.97E−03	0.36 ± 0.23	1.82E−03	0.23 ± 0.13	2.33E−03	0.39 ± 0.29
Chemokine	CXCL11	1.00E+00	1.67 ± 1.59	8.08E−05	5.1 ± 4.47	7.61E−04	4.32 ± 1.86	1.74E−03	4.86 ± 5.32
Chemokine	CXCL9	1.00E+00	1.67 ± 1.09	3.31E−08	6.12 ± 2.98	4.32E−04	4.65 ± 2.02	1.46E−05	5.33 ± 3.53
Chemokine	GROA	9.72E−02	3.7 ± 3.02	1.06E−08	69.55 ± 80.26	1.29E−04	81.98 ± 107.03	2.15E−08	51.8 ± 41.93
Chemokine	CCL7	1.00E+00	1.17 ± 0.54	7.50E−04	4.41 ± 3.62	1.00E+00	1.12 ± 0.35	2.37E−02	3.13 ± 2.32
Chemokine	CCL2	1.00E+00	1.06 ± 0.42	8.20E−02	2.41 ± 2.47	3.70E−02	1.93 ± 0.49	7.70E−02	2.72 ± 2.57
Chemokine	CXCL12	1.00E+00	1.11 ± 0.7	1.50E−03	1.7 ± 0.53	1.00E+00	1.15 ± 0.31	5.80E−01	1.34 ± 0.55
Chemokine	CXCL16	1.00E+00	0.95 ± 0.55	7.00E−04	0.12 ± 0.08	2.00E−03	0.09 ± 0.05	1.30E−01	0.35 ± 0.42
Chemokine	RANTES	1.00E+00	1 ± 0.34	1.00E+00	1.19 ± 0.26	1.00E+00	1.08 ± 0.14	4.00E−05	1.78 ± 0.56
Chemokine	IP10 (CXC10)	1.00E+00	1.11 ± 0.26	8.50E−06	1.84 ± 0.58	1.00E+00	1.23 ± 0.32	9.18E−08	2.53 ± 1.25
Chemokine	SCF	1.00E+00	1.62 ± 1.04	2.02E−07	5.32 ± 2.33	3.12E−02	3.3 ± 1.93	7.85E−03	3.37 ± 2.37
Inflam.cytok.	GM‐CSF	1.00E+00	0.79 ± 0.5	1.85E−06	33.74 ± 27.09	1.82E−04	25.4 ± 12.85	1.28E−04	20.75 ± 15.38
Inflam.cytok.	IL5	1.00E+00	1.29 ± 0.91	4.78E−04	2.48 ± 0.99	2.74E−03	2.53 ± 0.8	1.07E−03	2.45 ± 1.2
Inflam.cytok.	IL6	8.26E−01	2.1 ± 1.47	2.89E−10	46.55 ± 34.71	1.92E−04	17.25 ± 5.19	6.84E−08	38.04 ± 32.79
Inflam.cytok.	IL8	3.73E−01	2.72 ± 2.13	4.47E−08	13.37 ± 8.77	8.81E−03	6.49 ± 4.03	1.45E−08	13.29 ± 7.74
Inflam.cytok.	LIF	1.00E+00	1.09 ± 0.29	5.42E−08	7.01 ± 5.95	7.94E−03	2.2 ± 0.56	1.79E−03	3.83 ± 2.93
Inflam.cytok.	ST2	1.00E+00	0.9 ± 0.62	6.55E−08	0.22 ± 0.18	4.04E−06	0.18 ± 0.1	2.42E−04	0.36 ± 0.23
Inflam.cytok.	TWEAK	1.00E+00	1.45 ± 0.92	8.95E−04	2.65 ± 1.74	4.12E−02	2.23 ± 1.19	7.49E−04	2.78 ± 1.91
Inflam.cytok.	TNFA	3.73E−01	1.79 ± 1.27	1.30E−02	2.11 ± 0.94	2.00E−01	2.03 ± 1.19	1.15E−02	2.25 ± 1.3
Inflam.cytok.	IL17A	1.00E+00	1.07 ± 0.65	4.20E−03	1.92 ± 0.89	3.70E−01	1.45 ± 0.27	4.40E−02	1.55 ± 0.5
Inflam.cytok.	IL18	6.80E−01	1.92 ± 1.62	1.60E−02	2.4 ± 1.48	1.70E−01	2.32 ± 1.43	5.10E−02	2.2 ± 1.36
Inflam.cytok.	IL1A	1.00E+00	1.07 ± 0.28	1.00E+00	0.86 ± 0.28	3.30E−02	0.56 ± 0.35	1.00E+00	1.12 ± 0.52
Anti‐Inflam.	IL11	1.00E+00	1.36 ± 0.59	7.28E−07	22.7 ± 34.03	1.15E−05	42.04 ± 46.95	1.48E−03	4.97 ± 5.07
Anti‐Inflam.	IL4	1.00E+00	1.45 ± 0.61	2.15E−06	3.02 ± 1.19	1.10E−04	2.88 ± 0.91	6.31E−03	2.22 ± 1.04
Anti‐Inflam.	IL13	1.00E+00	1.03 ± 0.26	4.73E−06	1.88 ± 0.55	1.20E−01	1.37 ± 0.24	6.00E−04	1.62 ± 0.45
Anti‐Inflam.	IL22	1.00E+00	0.98 ± 0.18	1.65E−06	2.25 ± 0.85	6.40E−02	1.48 ± 0.21	5.24E−05	2.05 ± 0.84
ECM	MMP7	1.40E−04	2.03 ± 0.93	2.20E−03	1.69 ± 0.45	3.30E−01	1.48 ± 0.33	4.60E−04	1.82 ± 0.63
ECM	MMP1	8.39E−01	2.71 ± 2.95	4.11E−08	9.41 ± 3.89	6.26E−05	9.38 ± 5.2	1.31E−06	8.73 ± 5.34
Angio.Neuro	PROK1	7.42E−01	2.02 ± 1.47	1.51E−09	7.4 ± 4.08	1.14E−03	4.23 ± 1.26	1.71E−06	6.02 ± 4.11
Angio.Neuro	VEGF	1.00E−02	1.83 ± 0.73	1.56E−05	3.8 ± 2.94	6.30E−02	1.92 ± 0.85	1.50E−04	2.37 ± 1.16
Angio.Neuro	VCAM1	7.10E−02	2.1 ± 1.15	1.10E−03	4.19 ± 3.73	1.00E+00	1.07 ± 0.39	8.44E−08	5.07 ± 1.93
Growth factors	basic‐FGF	7.30E−01	1.96 ± 1.55	7.50E−04	3.34 ± 2.77	8.00E−01	1.86 ± 1.15	3.20E−03	3.58 ± 3.5
Growth factors	ICAM1	1.00E+00	1.2 ± 0.34	3.51E−06	2.58 ± 1.56	6.70E−01	1.22 ± 0.24	2.64E−05	2.27 ± 1.21
General	MCSF	1.17E−01	1.58 ± 0.65	2.00E−04	2.13 ± 0.85	2.70E−01	1.59 ± 0.54	2.00E−04	2.12 ± 0.85
General	FST	1.00E+00	1.14 ± 0.44	1.20E−02	0.56 ± 0.18	1.00E−04	0.32 ± 0.1	1.00E+00	1.08 ± 0.52
Regulator	IL9	1.00E+00	1.03 ± 0.65	1.50E−03	2.14 ± 1.12	1.00E+00	1.07 ± 0.36	1.96E−02	1.91 ± 0.97

*Note:* Fold.Change ± standard deviation: Relative expression against the untreated control followed by the associated standard deviation of the replicates. Green indicates significant up‐regulation, red indicates significant downregulation compared to untreated control.

Distinct expression patterns emerged for several additional markers under the different treatment conditions compared to untreated controls. Specifically, RANTES (CCL‐5) was uniquely upregulated by Pam2CSK4, highlighting its specific involvement in Pam2CSK4‐mediated inflammatory pathways (Figure 2D). CCL‐2 was significantly increased only following 1 ng/mL IL‐1β treatment, indicating a dose‐dependent effect of IL‐1β on this chemokine (Figure 2C, Table 1). Similarly, CXCL‐12 and IL‐18 were elevated exclusively in response to 100 pg/mL IL‐1β, further emphasizing the concentration‐dependent nature of IL‐1β's influence (Figure 2B, Table 1). CXC‐16 and FST showed a reverse related dose response to IL‐1β since higher concentrations of IL‐1β caused a reduction of the markers compared to lower concentrations of IL‐1β (Table 1). Moreover, MMP7 and VEGF were jointly upregulated by H_2_O_2_, 100 pg/mL IL‐1β and Pam2CSK4, suggesting some overlapping regulatory mechanisms (Table 1). Finally, some markers did have a Kruskall‐Wallis *p* value lower than 0.05 but the post hoc analysis revealed differences only between the stimulations or no significance between any condition.

### 3.4 IL‐6, IL‐8, GRO‐α, ICAM‐1, and MMP‐7 Were Secreted by Both IL‐1β and Pam2CSK4 Stimulated NP Cells in Alginate

The impact of Pam2CSK4 and 100 pg/mL IL‐1β on the NP cell secretome was also examined in an alginate bead culture system cultured under more physiological conditions of physioxia and low glucose conditions. The aim was to determine whether similarities or differential responses compared to cell responses in monolayer culture under standard high glucose and 21% O_2_ conditions could be identified.

Before proceeding with the differential secretion analysis, the baseline secretion of all markers and all donors was evaluated for a better understanding of donor variability in baseline secretion profiles (Figure S3). Baseline profiles were largely stable across donors. Anti‐inflammatory markers showed minimal variability, with TGF‐β1 relatively higher. In ECM, donor F was consistently highest; donors D and G were similar. For angiogenic/neurotropic factors, VEGF was higher across donors, PAI‐1 was elevated in donor F, and VCAM1 was lowest. Regulators clustered near ~0.3 pg/bead (low variability). Pro‐inflammatory cytokines were mostly 0.1–1 pg/bead, with MIF the highest. In chemokines, CCL19/20/22 and CXCL12/13/16 showed minimal variability (~10–100 pg/bead), whereas CCL17, CCL3, CXCL9, Eotaxin, GRO‐α, IL‐1Ra, IL‐8, IP‐10, RANTES, and SCF were lower (~0.1–1 pg/bead); CCL2, CCL4, and CXCL11 ranged 0.1–10 pg/bead. The proteins, b‐FGF, FST, and ICAM1 were similar, while M‐CSF remained low with minimal variability.

To determine significant differences in the secretome of NP cells cultured in 3D alginate following stimulation, a Kruskal–Wallis test identified 11 out of 73 protein markers as significantly different across treatments (*p* < 0.05; Table S6). Significantly altered markers included GRO‐α, IL‐6, IL‐8, ICAM1, TGF‐β1, b‐FGF, MMP‐7, MMP‐1, IL‐7, IP10, and CCL‐4. To further investigate these differences, group comparisons were conducted using Dunn's post hoc analysis with Bonferroni correction.

**FIGURE 3:**
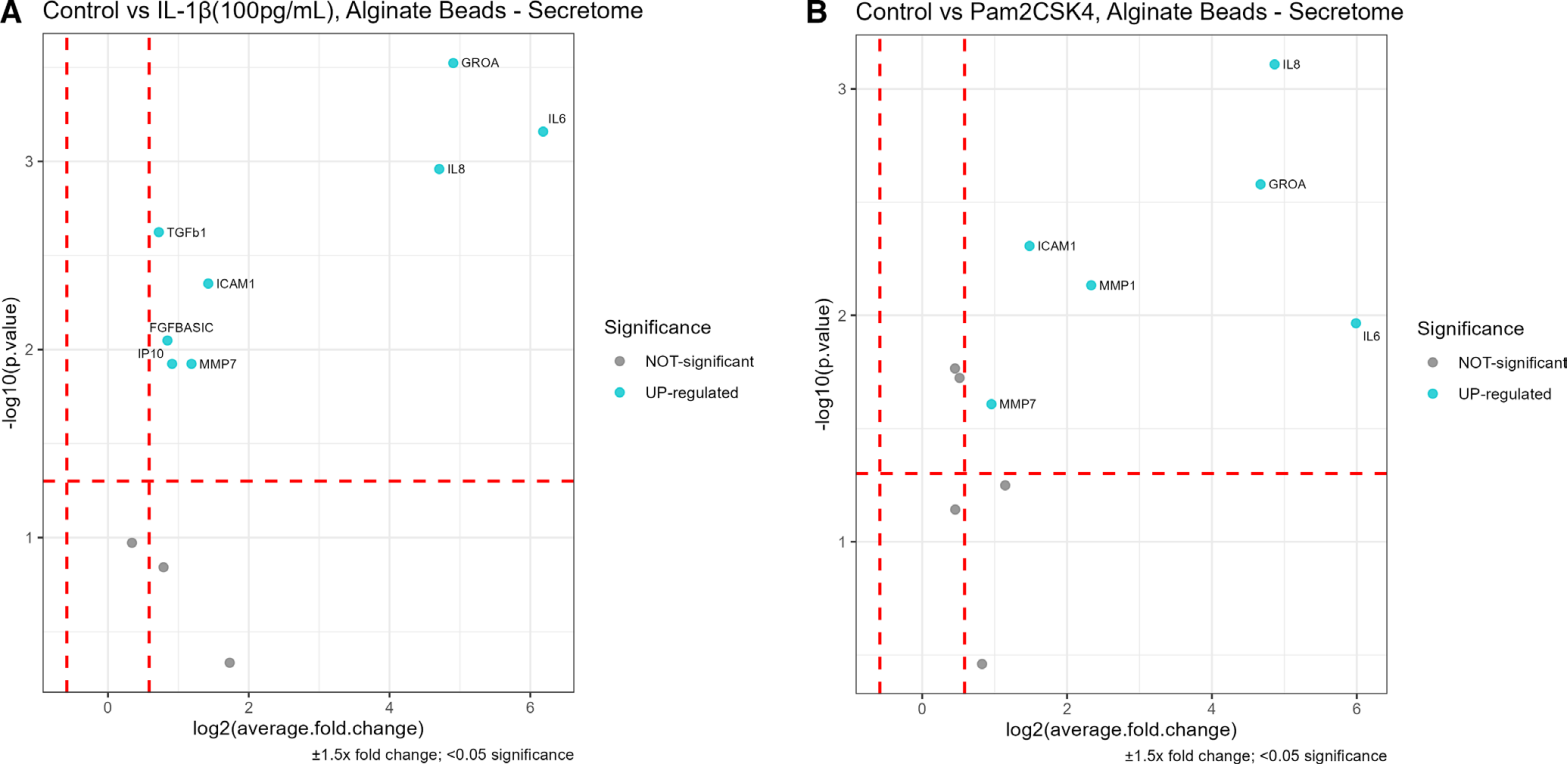
Differentially secreted markers after stimulating human NP cells with 100 pg/mL IL‐1β and 100 ng/mL Pam2CSK4 in alginate. (A) 100 pg/mL IL‐1β versus control, (B) Pam2CSK4 versus control. Group comparisons were performed using Dunn's post hoc analysis, with *N* = 3 and *n* = 3 for all stimulations. The plots display the comparisons between treated and untreated controls and the associated adjusted *p* value. Markers were deemed differentially expressed if their expression changed by ±1.5‐fold with *p* < 0.05 compared to the control. Protein expression for each marker was normalized to the total number of beads prior to the analysis.

The markers GRO‐α, ICAM‐1, IL‐6, IL‐8, and MMP‐7 were significantly elevated under both Pam2CSK4 and IL‐1β (100 pg/mL) treatments compared to untreated controls (Figure 3A,B, Table 2). Notably IL‐7 and b‐FGF did appear to be significantly different from the control in Pam2CSK4 treatment, but both had an average relative change compared to the control of less than 1.5; thus, they did not appear in the corresponding volcano plot (Table 2). MMP‐1 was differentially expressed only after the Pam2CSK4 treatment (Figure 3B), whereas TGF‐β1, IP10, and b‐FGF were uniquely upregulated in response to IL‐1β (Figure 3A). These findings suggest that while Pam2CSK4 and lower‐dose IL‐1β induce overlapping secretome changes, they also differentially modulate specific protein markers in NP cells within alginate bead cultures. Among the 11 markers, CCL‐4 did not reach significance in any pairwise comparisons (Table 2).

**TABLE 2:** Protein expression and statistical significance of NP cell secretions following IL‐1β and Pam2CSK4 stimulation in alginate cultures.

Aginate beads		IL‐1β (100 pg/mL) vs. control		Pam2CSK4 vs. control	
Type	Protein.Marker	Adj.*p*.value	Fold.change	Adj.*p*.value	Fold.change
Chemokine	CCL4	1.44E−01	1.73 ± 1.124	5.63E−02	2.21 ± 1.96
Chemokine	GROA	3.00E−04	29.97 ± 29.99	2.64E−03	25.49 ± 33.34
Chemokine	IL8	1.10E−03	26.11 ± 33.6	7.79E−04	29.15 ± 32.83
Chemokine	IP10	1.19E−02	1.88 ± 0.67	3.47E−01	1.77 ± 1.3
Inflam.cytok.	IL6	6.94E−04	72.62 ± 105.5	1.08E−02	63.5 ± 95.82
Inflam.cytok.	IL7	1.07E−01	1.27 ± 0.2	1.72E−02	1.37 ± 0.32
Growth factors	TGFb1	2.38E−03	1.65 ± 0.44	7.20E−02	1.37 ± 0.35
Growth factors	basic‐FGF	8.95E−03	1.8 ± 0.96	1.89E−02	1.43 ± 0.40
Growth factors	ICAM1	4.46E−03	2.68 ± 1.095	4.94E−03	2.79 ± 1.41
ECM	MMP1	4.62E−01	3.32 ± 4.22	7.36E−03	5.04 ± 4.66
ECM	MMP7	1.19E−02	2.28 ± 1.01	2.47E−02	1.94 ± 0.65

*Note:* Fold.Change ± standard deviation: Relative expression against the untreated control followed by the associated standard deviation of the replicates. Green indicates significant up‐regulation, whilst no significant down regualtion was identifed, compared to untreated control.

### 3.5 Comparison of Secretome Profiles After Pam2CSK4 and 100 pg/mL IL‐1β Stimulations in Monolayer and Alginate Cultures Indicated IL‐6, IL‐8, GRO‐α, ICAM‐1, and MMP‐7 as Consistently Secreted Factors

After examining the differential expression of secreted markers from NP cells stimulated with Pam2CSK4 and IL‐1β (100 pg/mL) in both monolayer and alginate cultures, fewer factors were secreted in 3D alginate under conditions of low glucose and physioxia than monolayer cultures in high glucose and high oxygen conditions. This observation underscores how the culture system and environmental conditions may influence the cellular secretome and the importance of utilizing culture systems which mimic more closely the native disc environment.

**FIGURE 4:**
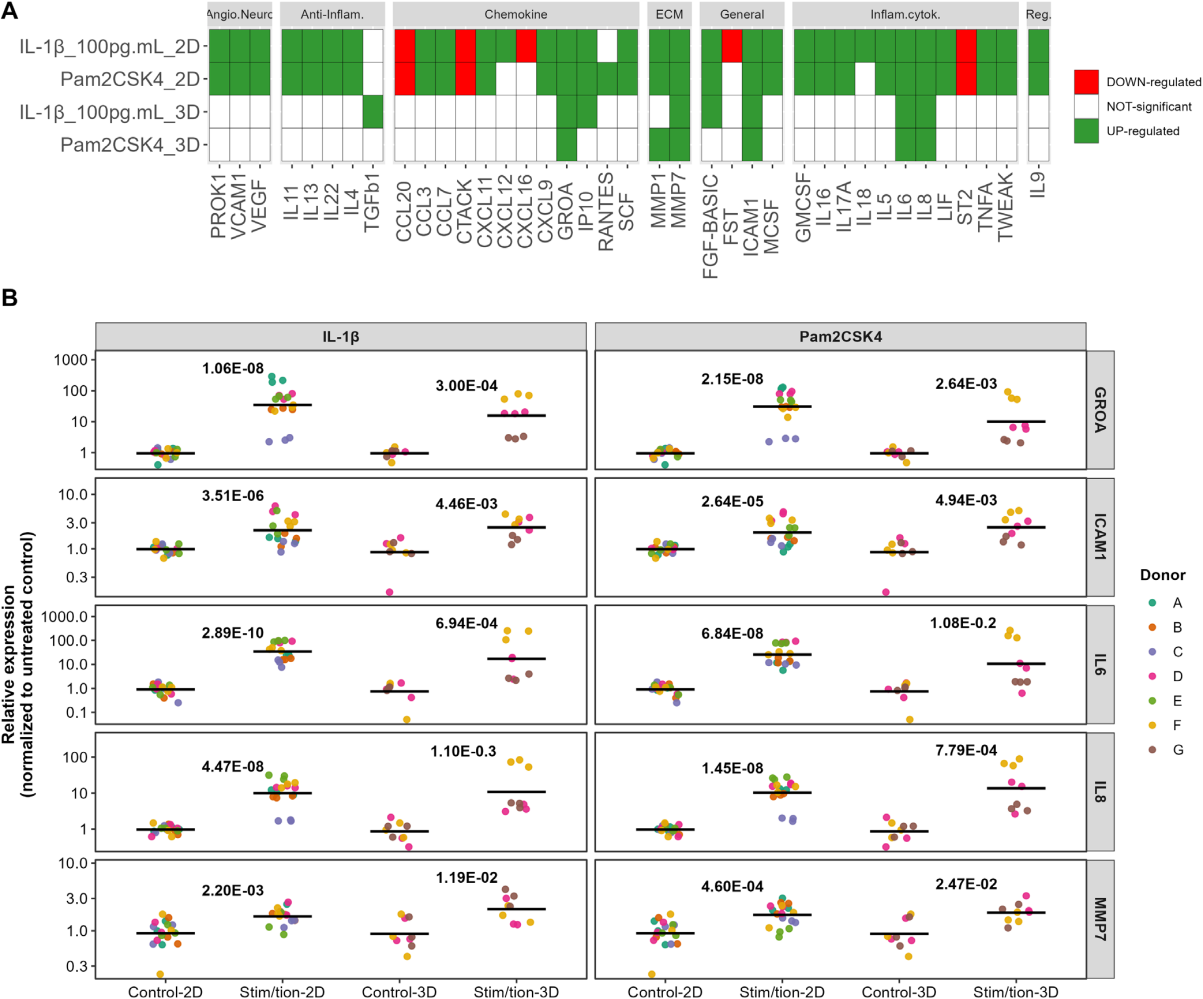
Marker regulation in monolayer and alginate. (A) Markers identified by differential secretion analysis in monolayer and alginate after stimulation with 100 pg/mL IL‐1β or 100 ng/mL Pam2CSK4, (B) expression of the markers GRO‐α, ICAM1, IL‐6, IL‐8, and MMP‐7 in monolayer and alginate after stimulation with 100 pg/mL IL‐1β or 100 ng/mL Pam2CSK4. The *p* value shown is the adjusted *p* value after the multiple comparison test in the differential secretion analysis step.

MMP‐1 was upregulated in monolayer under both IL‐1β and Pam2CSK4 stimulation, whereas in alginate it was only elevated by Pam2CSK4 (Figure 4A). TGF‐β1 was differentially expressed exclusively in response to IL‐1β (100 pg/mL) in alginate cultures (Figure 4A). IP10 and b‐FGF were induced by both treatments in monolayer, but in the alginate system were upregulated following only IL‐1β (100 pg/mL) stimulation (Figure 4A). PROK1, VCAM1, VEGF, IL‐11, IL‐13, IL‐22, IL‐4, CCL‐20, CCL‐3, CCL‐7, CTACK, CXCL‐11, CXCL‐9, SCF, M‐CSF, GM‐CSF, IL‐16, IL‐17A, IL‐5, LIF, ST2, TNFA, TWEAK, and IL‐9 were differentialy expressed in monolayer by both stimulations (Figure 4A). CXCL‐12, CXCL‐16, FST, and IL‐18 were differentially secreted only after 100 pg/mL IL‐1β in monolayer (Figure 4). RANTES was differentially secreted only after 100 ng/mL Pam2CSK4 in monolayer. Among all the markers significantly altered compared to untreated controls in both culture systems IL‐6, IL‐8, GRO‐α, MMP‐7, and ICAM‐1 were consistently altered relative to the control in both culture systems following both treatments (Figure 4B).

### 3.6 Pam2CSK4 and IL‐1β Stimulations Are Characterized by a Similar Phosphoproteomic Response in Monolayer Culture

**FIGURE 5:**
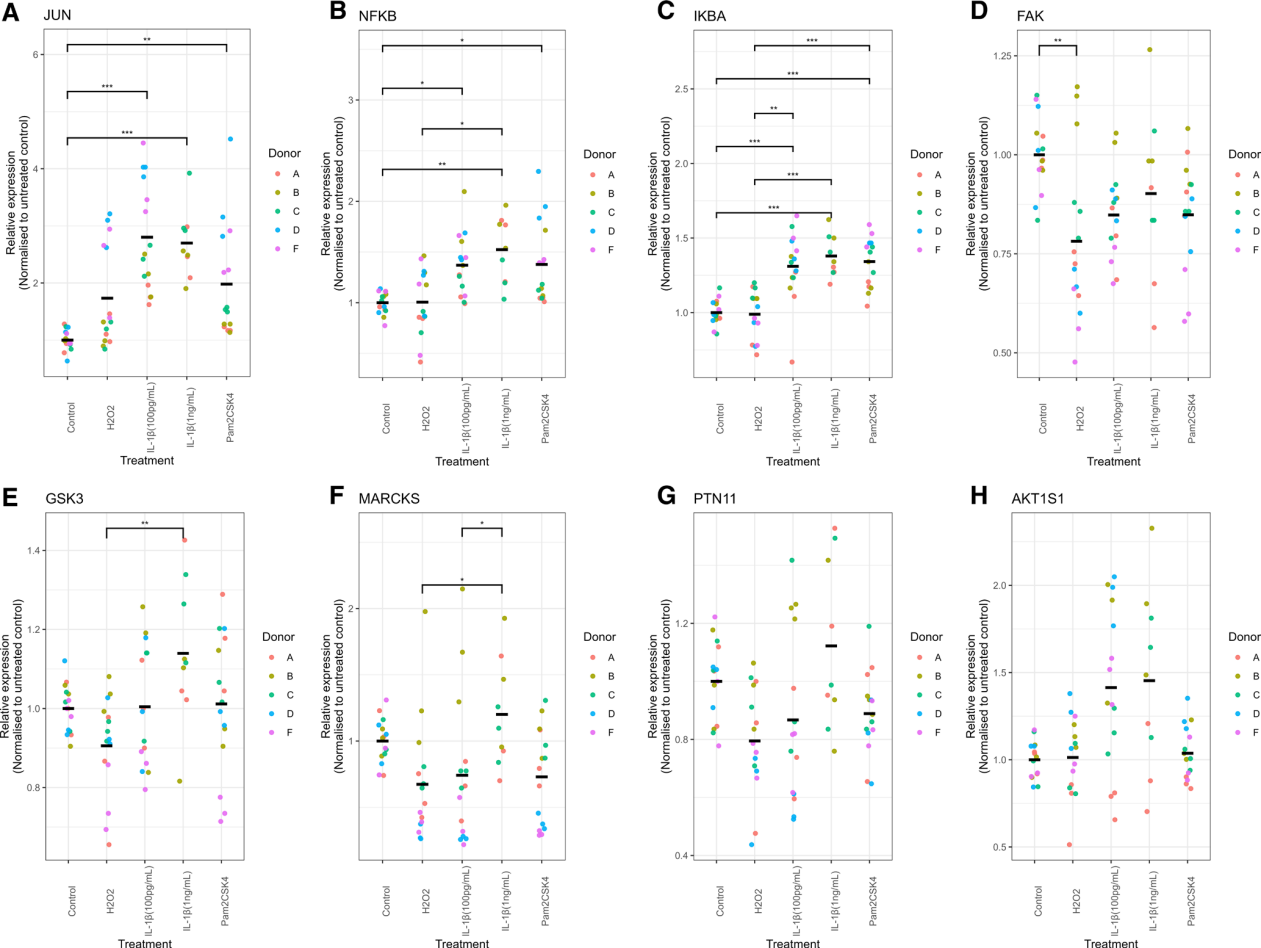
IL‐1β, H_2_O_2_, and Pam2CSK4 induced phospho‐proteome profiles on human NP cells. (A) JUN, (B) NF‐kB, (C) IKBA, (D) FAK, (E) GSK3, (F) MARCKS, (G) PTN‐11, (H) AKT1S1. Each dot represents a replicate normalized to the average of the untreated controls of the same donor. Kruskal–Wallis test and a Dunn's multiple comparisons post hoc test rank‐sum statistical test was performed with a *p* value < 0.05, which was considered statistically significant (*N* = 3–5 and *n* = 3, *p* > 0.05: Not significant, *p* < 0.05 and *p* > 0.01: *, *p* < 0.01 and *p* > 0.001: **, *p* < 0.001: ***). The average of the points is shown for each treatment.

In the final stage of the proteomic investigation, signaling pathways associated with the secretome profiles of NP cells under various stimulations in monolayer cultures were identified using a 21‐plex assay. A Kruskal–Wallis test revealed that p‐JUN (*p* < 0.001), p‐NF‐kB (*p* < 0.001), p‐IkBα (p < 0.001), Phosphorylated Focal Adhesion Kinase (p‐FAK) (*p* = 0.004), Phosphorylated Glycogen Synthase Kinase 3 (p‐GSK3) (*p* = 0.022), Phosphorylated Myristoylated Alanine‐Rich C Kinase Substrate (p‐MARCKS) (*p* = 0.0023), Phosphorylated Phosphotyrosine‐Protein 11 (p‐PTN11) (*p* = 0.011) and Phosphorylated AKT Serine/Threonine Kinase 1 Subunit 1 (p‐AKT1S1) (*p* = 0.016) were significantly altered across treatments. Subsequent Dunn's multiple comparison post hoc analysis revealed significant differences between groups (Table 3) for all the markers except PTN11 (Figure 5G) and AKT1S1 (Figure 5H).

**TABLE 3:** Phosphorylation responses of NP cells to Pam2CSK4, H_2_O_2_, and IL‐1β in monolayer culture.

Monolayer	H2O2 vs. control		IL‐1β(100pg/mL) vs. control		IL‐1β(1ng/mL) vs. control		Pam2CSK4 vs. control	
Protein.marker	Adj.*p*.value	Fold.change	Adj.*p*.value	Fold.change	Adj.*p*.value	Fold.change	Adj.*p*.value	Fold.change
AKT1S1	1.00E + 00	1.01(+/−)0.23	9.60E−02	1.41(+/−)0.47	2.20E−01	1.45(+/−)0.52	1.00E+00	1.04(+/−)0.16
IKBA	1.00E + 00	0.99(+/−)0.16	5.60E−04	1.31(+/−)0.23	3.10E−04	1.38(+/−)0.14	1.90E−04	1.34(+/−)0.17
FAK	2.04E−03	0.78(+/−)0.21	5.70E−02	0.85(+/−)0.11	1.00E+00	0.9(+/−)0.21	9.00E−02	0.85(+/−)0.14
GSK3	8.40E−01	0.91(+/−)0.13	1.00E+00	1(+/−)0.15	6.80E−01	1.14(+/−)0.18	1.00E+00	1.01(+/−)0.18
JUN	1.08E−01	1.73(+/−)0.89	1.00E−06	2.8(+/−)0.96	5.46E−05	2.7(+/−)0.59	7.46E−03	1.98(+/−)0.99
MARCKS	5.40E−02	0.67(+/−)0.45	8.00E−02	0.74(+/−)0.56	1.00E+00	1.2(+/−)0.41	4.60E−01	0.73(+/−)0.36
PTN11	6.40E−02	0.79(+/−)0.19	2.60E−01	0.87(+/−)0.29	1.00E+00	1.12(+/−)0.29	1.00E+00	0.89(+/−)0.14
NFKB	1.00E + 00	1.01(+/−)0.33	1.10E−02	1.37(+/−)0.31	1.90E−03	1.52(+/−)0.33	2.80E−02	1.38(+/−)0.4

*Note:* Fold.Change ± standard deviation: Relative expression against the untreated control followed by the associated standard deviation of the replicates. Green indicates significant up‐regulation, whilst no significant down‐regulation was observed, compared to untreated control.

IL‐1β increased multiple targets in relation to the untreated control: at 1 ng/mL, IκBα (Figure 5C, *p* < 0.001), c‐Jun (Figure 5A, *p* < 0.001), and NF‐κB (Figure 5B, *p* = 0.0019); at 100 pg/mL, IκBα (Figure 5C, *p* < 0.001), c‐Jun (Figure 5A, *p* < 0.001), and NF‐κB (Figure 5B, *p* = 0.011). Similarly, Pam2CSK4 increased IκBα (Figure 5C, *p* < 0.001), c‐Jun (Figure 5A, *p* = 0.0075), and NF‐κB (Figure 5B, *p* = 0.028) relative to untreated controls. H_2_O_2_ decreased FAK phosphorylation (Figure 5D, *p* = 0.002) relative to controls. Finally, the expression of GSK3 and MARCKS was lower in H_2_O_2_ treated cells compared to 1 ng/mL IL‐1β (Figure 5E, *p* < 0.001; Figure 5F, *p* < 0.05) while dose dependence was observed for MARCSK after IL‐1β treatment (Figure 5F, *p* < 0.05) (Table 3).

## 4 Discussion

This study aimed to determine the in vitro molecular responses of human NP cells to stressors relevant to IDD, namely to the plethoric cytokine: IL‐1β, a TLR2 agonist: Pam2CSK4 to model potential responses from PAMPS and DAMPS within the degenerate disc, and H_2_O_2_ to model the influence of oxygen free radicals. These stimulations were performed within two key culture systems and environmental conditions: (1) standard monolayer culture under standard high glucose culture media in 21% O_2_; (2) 3D alginate culture following a period of culture to enable re‐differentiation to an NP phenotype and cultured within a closer physiological environment to the native disc of low glucose serum free media under physioxia. These culture systems represent (1) the most commonly applied culture system utilized for NP cells within research labs across the world [21], and (2) an environment which mimics more closely that of the native disc and was recommended in the recent ORS Spine section initiative for harmonization of culture methodology [21]. In vitro molecular responses were determined using Luminex‐based immunoassays, to determine a broad characterization of secretomics and phosphoproteomics characterizing cellular signaling processes. Importantly this study identified a rich secretome produced by human NP cells, with higher production rates observed in monolayer, high glucose, and high oxygen conditions compared to 3D culture within low glucose and low oxygen conditions. The stimulation with stressors relevant to IDD identified five protein markers, namely IL‐6, IL‐8, GRO‐α, ICAM1, and MMP‐7 that were consistently upregulated across both monolayer and alginate cultures following stimulation with IL‐1β and Pam2CSK4, furthermore both factors upregulated the intracellular markers p‐cJUN, p‐NF‐κB and p‐IkBα within monolayer cultures. Additionally, distinct differences in secreted markers were observed between IL‐1β and Pam2CSK4 stimulations and culture conditions. Hydrogen peroxide primarily reduced cell viability at a high concentration while NP cells treated with sub toxic doses had limited effects on the NP cell secretome or signaling pathways.

IL‐6, IL‐8, GRO‐α, ICAM‐1, and MMP‐7 were identified as commonly secreted factors, suggesting their importance in IDD. IL‐6 acts as a potent pro‐inflammatory cytokine associated with back pain and perpetuates the catabolic effects of IL‐1β within the disc environment [6]. It has been shown that degenerated discs have increased levels of IL‐8 compared to non‐degenerated ones and also that the protein is elevated in the cerebrospinal fluid (CSF) as well as degenerating disc tissues of individuals with chronic LBP [7, 27]. Elevated serum levels of IL‐6 and IL‐8 are associated with long‐lasting lumbar radicular pain following disc herniation, suggesting their role in the underlying inflammatory processes and potential as biomarkers for chronic pain prognosis [28]. GRO‐α (CXCL‐1) is a chemokine of the C‐X‐C subfamily that is secreted during inflammatory processes and has been detected at elevated levels in degenerated IVDs [7]. CXCL‐1 binds to and activates the CXCR‐2 receptor, a pathway reported to drive and exacerbate radicular pain [29]. Meanwhile, MMP‐7 can compromise the structural integrity of the disc by degrading the ECM and has been found to be elevated in degenerated discs [30]. ICAM‐1 is a cell surface protein that facilitates intercellular communication. A study by Gabr et al. showed that when disc cells from AF or NP in culture are exposed to IL‐17 in combination with TNF or IFN‐γ, then ICAM‐1 expression is increased [31]. This observation coincides with current findings in monolayer since IL‐17A and TNF were also differentially secreted after 100 pg/mL IL‐1β or 100 ng/mL Pam2csk4. Collectively, these markers create a pro‐inflammatory and catabolic state, with angiogenic potential thus underscoring their importance as targets for therapeutic interventions aiming to mitigate the degenerative changes characteristic of IDD. These factors may function as early drivers of inflammatory and catabolic events in the disc, potentially setting the stage for the progression of disc degeneration.

TGF‐β1, a growth factor associated with matrix synthesis and protective IVD effects, was differentially secreted only in alginate after stimulation with 100 pg/mL IL‐1β showcasing the importance of in vitro models in the study of disc degeneration [32]. The expression of this marker could also explain significantly smaller numbers of secreted factors in alginate since it is reported to be able to block pro‐inflammatory pathways that could be initiated by autocrine signaling [33]. The number of markers expressed in monolayer in which the cells had a high amount of glucose, FCS, and oxygen was considerably higher compared to alginate culture which was cultured under conditions of low glucose, no FCS, and low oxygen concentration. This is particularly relevant, as studies have shown that different types of fetal bovine serum (FBS) can cause variance in IL‐8 secretion by epithelial cells, likely due to small molecule metabolites affecting the ERK pathway [34]. Consistent with field practice, our 2D monolayer experiments were conducted in high‐glucose media at 21% O_2_, whereas 3D alginate re‐differentiation used low‐glucose DMEM at 5% O_2_ [21]. The NP culture harmonization study documents that most laboratories expand NP cells in high‐glucose media at 17%–21% O_2_ and then switch to low‐glucose DMEM and ~5% O_2_ during 3D re‐differentiation to better approximate the avascular, low‐oxygen NP niche and maintain phenotype [21]. These environmental parameters are therefore expected to modulate the magnitude of secretory responses and likely contributed to the broader secretome we observed in 2D [35]. Monolayer culture captures a de‐differentiated NP‐cell state that tends to produce a broader inflammatory and catabolic secretome in response to IL‐1β and TLR2/6 [36]. In contrast, 3D alginate supports redifferentiation and matrix production. Importantly, however, five core mediators (IL‐6, IL‐8, GRO‐α, ICAM‐1, MMP‐7) were induced by IL‐1β/Pam2CSK4 in both environments, indicating that the principal stimulus‐dependent pathways are robust across model systems. Using two standard, complementary culture formats was intended to compare treatment effects on the secretome across a de‐differentiated 2D context and a re‐differentiated 3D context as we did not vary oxygen or glucose factorially. Future work explicitly decoupling O_2_/glucose from dimensionality will be valuable for attributing effects to individual microenvironmental cues.

IL‐1β at 100 pg/mL in alginate and monolayer as well as Pam2CSK4 in monolayer caused the differential expression of IP10 and b‐FGF. IP10 (CXCL10) interacts with immune cells, including T lymphocytes and monocytes, by binding to the CXCR‐3 receptor expressed on their surfaces, thereby facilitating their recruitment and activation during inflammatory responses [37]. B‐FGF plays a dual role in IVD and articular cartilage homeostasis by promoting cell growth and ECM synthesis while also upregulating catabolic enzymes and inhibiting matrix formation, highlighting its complex involvement in disc degeneration and the need for further research to elucidate its therapeutic potential [38]. Lastly, MMP‐1 was upregulated by Pam2CSK4 in alginate and monolayer and by IL‐1β only in monolayer. Deng et al. showed that MMP‐1 levels were significantly elevated in both the serum and IVD tissues of IDD patients, particularly in severe cases, highlighting its essential role in ECM degradation and the progression of IDD [39].

Although most IVD studies emphasize upregulated mediators, our 2D monolayer experiments identified a small set of downregulated proteins after IL‐1β and/or Pam2CSK4: CCL‐20, CCL‐27/CTACK, and ST2 (decreased with both stimuli), plus CXCL‐16, Follistatin (FST), and IL‐1α (decreased with IL‐1β). In the IVD context, these reductions are informative when viewed alongside the concurrent increases we observed in IL‐6, IL‐8, GRO‐α, ICAM‐1, and MMP‐7 (core set elevated in both 2D and 3D), and broader monolayer‐only increases in pro‐inflammatory and catabolic factors. Together, the pattern supports a rich inflammatory secretome in NP cells that prioritizes potential chemoattraction and matrix catabolism. The Luminex assay detects soluble ST2, a decoy receptor for IL‐33. Its decrease would be expected to lessen IL‐33 neutralization, potentially permitting greater IL‐33 bioactivity aligning with reports linking the IL‐33/ST2 axis to radicular pain in disc disease and with the pro‐algesic secretome we observed (elevated IL‐6/IL‐8/GRO‐α) [40]. While IL‐33 itself was not measured here, the concordance between lowered ST2 and a pro‐inflammatory secretome is biologically plausible in IVD. CCL‐20 produced by NP cells contributes to Th17‐cell trafficking into degenerated discs [41] In our monolayer data, CCL‐20 decreased despite IL‐17A and TNF rising under 100 pg/mL IL‐1β and Pam2CSK4, while IL‐8 and GRO‐α markedly increased. This decoupling suggests that acute IL‐1/TLR2/6 stimulation in NP cells preferentially amplifies neutrophil/monocyte chemotaxis (CXCR1/2 ligands) over CCR6‐dependent Th17 recruitment—an innate‐skewed inflammatory phenotype consistent with early degenerative signaling. A skin‐homing chemokine, CCL‐27 is not central to disc inflammation; its reduction under IL‐1/TLR2/6 stimulation supports the view that NP cells de‐emphasize this axis during inflammatory activation [42]. Evidence for CXCL‐16 in IVD is limited; however, reduced soluble CXCL‐16 under IL‐1β suggests the CXCR6 axis is not a major component of the NP response to these stimuli, in contrast to robust CXCR1/2‐ligand induction (IL‐8, GRO‐α) [43]. As our assay quantifies the soluble chemokine (the membrane‐tethered form was not measured), additional studies will be needed to define CXCL‐16 biology in NP cells. FST antagonizes activins with anti‐inflammatory and matrix‐protective effects reported in joint tissues. Its decrease following IL‐1β would remove an anti‐activin brake, consistent with the catabolic milieu we detected (elevated IL‐6, MMP‐7) and supporting the concept that boosting FST/anti‐activin signaling could be protective in the disc [44]. Despite IL‐1α's catabolic potential in the disc, we observed a dose‐dependent decrease with 1 ng/mL IL‐1β, possibly reflecting isoform cross‐regulation at the IL‐1R pathway and/or compensatory increases in other cytokines (e.g., IL‐4/IL‐11) evident in our monolayer profiles [45]. Net catabolic signaling remained strongly positive given the broad IL‐1β/TLR‐driven increases in IL‐6, IL‐8, GRO‐α, and MMP‐7 [20].

Other proteins upregulated in monolayer cultures following IL‐1β and Pam2CSK4 stimulations that have been linked to disc degeneration included: CCL‐3, CXCL‐11, CXCL‐9, LIF, IL‐11, MMP1, IL‐4, IL‐16, IL‐5, and GM‐CSF [6, 7, 46]. In contrast, the roles of PROK1, SCF, and TWEAK (TNF12) in disc degeneration remain unclear and need further investigation. Markers that were upregulated only after 100 pg/mL IL‐1β and Pam2CSK4 stimulation were CCL‐7, b‐FGF, IL‐13, IL‐17A, IL‐22, IL‐9, IP10, M‐CSF, TNF, and VCAM1, with most of them associated with disc degeneration or arthritis [7, 9, 45, 46, 47, 48]. Finally, CCL‐2 was differentially secreted after 100 pg/mL IL‐1β, and RANTES (CCL‐5) was upregulated after Pam2CSK4 stimulation, both of which have been shown to be elevated in degenerated discs [7]. CXCL‐12 together with IL‐18 was differentially expressed after 1 ng/mL IL‐1β and has been associated with IDD [7, 49].

Across assays, IL‐1β exhibited clear dose sensitivity. At the secretome level, several factors were induced only at the lower dose (100 pg/mL), for example CCL‐2, whereas others such as IL‐18 and CXCL‐12 appeared only following 1 ng/mL stimulation, and MMP‐2 and DEFB1 differed significantly between the two IL‐1β doses (Table 1; Figure 2B,C). We also noted a dose‐dependent decrease in IL‐1α protein levels with increasing IL‐1β suggesting engagement of negative‐feedback circuits [50]. At the phosphoprotein level, p‐MARCKS was lower at 100 pg/mL versus 1 ng/mL IL‐1β, indicating non‐linear regulation of PKC/MARCKS‐dependent cytoskeletal/exocytic processes with increasing IL‐1 receptor activation. Taken together, these data imply that “low‐grade” IL‐1β (100 pg/mL) is sufficient to prime chemokine‐rich inflammatory outputs, whereas higher IL‐1β (1 ng/mL) can shift the profile by additionally recruiting factors like IL‐18/CXCL‐12 and altering intracellular effectors such as MARCKS [51]. This non‐monotonicity is consistent with the broader cytokine literature in which receptor occupancy and induction of inhibitory loops shape concentration‐ and time‐dependent responses [52, 53]; in our dataset, these dynamics are reflected by the specific marker sets that diverge across doses.

Phosphoproteomic mapping showed that both IL‐1β (100 pg/mL and 1 ng/mL) and the TLR2/6 agonist Pam2CSK4 converged on a shared MyD88‐dependent inflammatory network, evidenced by significant increases in p‐IκBα, p‐NF‐κB, and p‐cJUN [54, 55]. In contrast, 50 μM H_2_O_2_ did not elicit this canonical inflammatory phospho‐signature and instead selectively reduced p‐FAK, consistent with ROS‐mediated perturbation of integrin/FAK signaling and cell adhesion/survival programs [56]. We also observed stimulus‐ and dose‐sensitive differences at other nodes (e.g., p‐GSK3 and p‐MARCKS differed between H_2_O_2_ and high‐dose IL‐1β; p‐MARCKS was lower at 100 pg/mL than 1 ng/mL IL‐1β), indicating modulation of cytoskeletal/exocytic and glycogen‐synthase‐kinase axes alongside NF‐κB/AP‐1. Functionally, these pathway signatures align with the robust secretion of IL‐6, IL‐8, GRO‐α, ICAM‐1, and MMP‐7 that we detected across both culture systems with IL‐1β and Pam2CSK4, whereas H_2_O_2_ primarily impacted viability and favored angiogenic/remodeling cues (e.g., VEGF, MMP‐7) with comparatively muted inflammatory signaling [57].

Stimulation with H_2_O_2_ primarily compromised cell viability at high concentrations and only modestly affected secretome or phosphoprotein profiles. This suggests that OS may not induce the same robust inflammatory signaling as classical pro‐inflammatory stimuli. Instead, H_2_O_2_ may accelerate degenerative processes primarily by damaging cellular components and promoting angiogenesis since VEGF and MMP‐7 were differentially expressed in monolayer. Interestingly, while previous studies have reported increased expression of senescence markers such as p16^INK4A^ or p21^WAF1^ following similar stressors, our findings did not show significant changes in these markers under any of the tested conditions [18, 58, 59]. This discrepancy could be attributed to differences in experimental design, particularly the duration of stimulation. Unlike other studies that limited stimulation to a few days, the experiments of the current study were extended to 1 week, potentially allowing NP cells to adapt or recover from the stress. Furthermore, when investigating the baseline expression of markers in untreated controls it was observed that high baseline expression of markers could have affected the responsiveness to treatments which could be an issue when establishing senescent models since cells from some donors might have adapted to stress while others do not. Interestingly this stimulation was the only one that reduced the marker FAK (Focal Adhesion Kinase 1), which is a non‐receptor tyrosine kinase that regulates cell adhesion, mechanotransduction, migration, and survival by linking ECM signals to intracellular pathways [60]. In osteoarthritis (OA), FAK is overactivated in chondrocytes, promoting cartilage degradation, inflammation, and abnormal cell signaling, contributing to the progression of joint degeneration [60]. Thus, even though H_2_O_2_ didn’t seem to regulate molecules related to disc degeneration its role needs in disease establishment and progression needs more research.

To our knowledge, this is the first study in primary human NP cells to pair a 73‐plex secretome with a 21‐plex phosphoprotein panel across disease‐relevant stimuli IL‐1β (two doses), TLR2/6 agonism, and OS, and across both monolayer and 3D re‐differentiated alginate culture. This design revealed shared and stimulus‐specific responses and yielded a concise, reproducible five‐marker panel (IL‐6, IL‐8, GRO‐α/CXCL‐1, ICAM‐1, MMP‐7) that was consistent across 2D and 3D culture. The phosphorylation data show convergence on NF‐κB/AP‐1 signaling, reinforcing a central inflammatory pathway. Together, these readouts provide practical endpoints for comparative studies and potency‐style assays during therapy development.

Practically, the five‐marker panel, paired with the NF‐κB/AP‐1 signature, offers translatable readouts of NP activation for screening and dose–response studies, potency testing of biologic/cell/gene candidates, and exploratory biomarker work in disc explants or in vivo models. Because these signals increased across stimuli and in both culture formats, they form a focused, mechanism‐linked set to track target engagement (e.g., IL‐1R or TLR2/6 blockade; IKK or JNK inhibition) and quantify treatment effects ahead of clinical translation.

## 5 Study Limitations

Firstly, the small donor pool restricts the generalizability of these results as well as the ability to draw definitive conclusions. Larger‐scale studies and more physiologically relevant disc models will be needed to validate these observations and to refine our understanding of NP cell pathobiology. Second, we did not perform 3D experiments under normoxia/high‐glucose. This intentional design choice follows consensus workflows (high‐glucose, 17%–21% O_2_ for expansion; low‐glucose DMEM, ~5% O_2_ in 3D) and enables comparison of stimulus responses across culture conditions. However, the culture design did not enable differences to be attributed to differences in monolayer v/s alginate culture, low versus high glucose and low versus high oxygen concentrations. Third, the use of multiple treatments per experiment reduced statistical power, potentially masking significant differences under stringent corrections. Comparisons that appeared significant when examining only two conditions lost significance upon inclusion of multiple treatments. This phenomenon highlights the challenges posed by multi‐group analyses, where strict statistical corrections reduce power. From a translational perspective, such observations emphasize that targeted pairwise comparisons may sometimes detect meaningful differences that are obscured when broader analyses are performed. Fourth, the concentrations and duration of H_2_O_2_ exposure used here may not fully capture the role of OS in the chronic setting of IDD. Finally, endpoint measurements were taken at 1 week, which may have overlooked earlier transient or later sustained responses.

The findings also demonstrated notable inter‐donor variability, particularly in baseline marker expression and the magnitude of response. Some donors showed high baseline levels of key mediators, while others had minimal changes in the same conditions. This heterogeneity underscores the clinical complexity of IDD, where therapeutic interventions may need to account for patient‐specific profiles. Such variability highlights the importance of replicating experiments with cells from multiple donors to capture a realistic spectrum of responses.

Finally, this work, whilst identifying pathway upregulation and downstream secretomic production, did not target functional assays to isolate individual mechanistic pathways. To fully elucidate functional mechanisms, receptor or kinase inhibition, gene knockdown, or rescue experiments would be required.

## 6 Conclusion

These results underscore the intricate and highly dynamic nature of NP cell responses to both inflammatory and oxidative stimuli. Notably, IL‐1β and Pam2CSK4 were found to consistently upregulate a common set of key mediators IL‐6, IL‐8, GRO‐α, ICAM1, and MMP‐7 across different culture conditions. This consistent activation highlights their potential role as core effectors in stress‐induced disc degeneration. While the culture environment (monolayer/high glucose/high oxygen vs. alginate/low glucose/low oxygen) influenced the breadth and magnitude of the secretome response, the strong overlap in inflammatory signatures between conditions suggests that fundamental pathways are conserved. In contrast, H_2_O_2_ exposure had more modest effects, suggesting a distinct, non‐secretome mediated mechanism of action.

Together, these findings offer a valuable molecular framework for understanding NP cell pathophysiology and support the use of IL‐6, IL‐8, GRO‐α, ICAM1, and MMP7 as candidate markers for further elucidation on their role in IDD. Furthermore, the data advocates that the cellular microenvironment and stimulus‐specific effects should be carefully considered before any study. Importantly, the use of high‐throughput multiplex immunoassays in this study enabled a broad and parallel profiling of both extracellular and intracellular responses, offering a detailed systems‐level view of NP cell behavior under stress. This comprehensive approach, along with phosphoproteomic validation, provides new insights into the convergence of inflammatory and catabolic signaling pathways that drive disc degeneration.

## Author Contributions

L.G.A., C.L.L.M., and E.K. performed the conceptualization of the study. E.K. ran the main experiments, executed the Luminex measurements, wrote the main text, performed the data analysis, and prepared the visualizations and graphs. A.N. and J.S. contributed with cell culture. PCR experiments, and data collection. L.G.A. and C.L.L.M. supervised the research, reviewed and edited the manuscript, and sourced funding. All authors approved the final version of the manuscript.

## Funding

Financial support was received from the Training network to advance integrated computational simulations in translational medicine, applied to intervertebral disc degeneration #955735 (https://cordis.europa.eu/project/id/955735).

## Conflicts of Interest

E.K. and L.G.A. were employed by PROTAVIO Ltd. All other authors declare no conflicts of interest.

## Supporting information


**Figure S1:** Human NP cell viability after treatment with different concentrations of H_2_O_2_. Viability was estimated using the ratio of treated samples to untreated controls (*n* = 5, *p* > 0.05: not significant, *p* < 0.05 and *p* > 0.01: *, *p* < 0.01 and *p* > 0.001: **, *p* < 0.001: ***).
**Figure S2:** Baseline levels of secreted markers by unstimulated human NP cells after 7 days in monolayer. Protein levels were first normalized to the total protein levels of the corresponding well and then the three replicates of each donor were normalized to their average expression.
**Figure S3:** Baseline levels of secreted markers by unstimulated human NP cells after 7 days in alginate. Protein levels were first normalized to the number of beads of the corresponding well and then the three replicates of each donor were normalized to their average expression.

**Data S1:** Tables S1–S9 provide supporting metadata, assay details, raw/processed outputs, quality control, and statistical analyses for the study. Specifically, it contains donor information (S1), RT–PCR gene/assay details (S2), the marker lists for the 73‐plex secretome and 21‐plex phosphoproteomic panels (S3–S4), phosphoproteomic QC results (S5), Kruskal–Wallis statistical outputs comparing monolayer, alginate, and phosphoproteomic datasets (S6), quantitative secretome concentrations (ng/mL) and phosphoproteomic readouts (Net MFI) (S7–S8), and a sample ID key together with the supplementary figures (S9).

## Data Availability

The data that supports the findings of this study are available in the Supporting Information of this article.
