# Supplementary material for: Secretome and Pathway Analysis of Stress Induced Disc Degeneration In Vitro
Source: JOR Spine. 2026 Mar 27;9(2):e70165. doi: 10.1002/jsp2.70165 (PMC13238643; doi:10.1002/jsp2.70165)
Supplement: Supplementary file 1 — Figure S1: Human NP cell viability after treatment with different concentrations of H2O2. Viability was estimated using the ratio of treated samples to untreated controls (n = 5, p > 0.05: not significant, p < 0.05 and p > 0.01: *, p < 0.01 and p > 0.001: **, p < 0.001: ***). Figure S2: Baseline levels of secreted markers by unstimulated human NP cells after 7 days in monolayer. Protein levels were first normalized to the total protein levels of the corresponding well and then the three replicates of each donor were normalized to their average expression. Figure S3: Baseline levels of secreted markers by unstimulated human NP cells after 7 days in alginate. Protein levels were first normalized to the number of beads of the corresponding well and then the three replicates of each donor were normalized to their average expression. [file JSP2-9-e70165-s002.docx]

**SUPPLEMENTARY FIGURES**


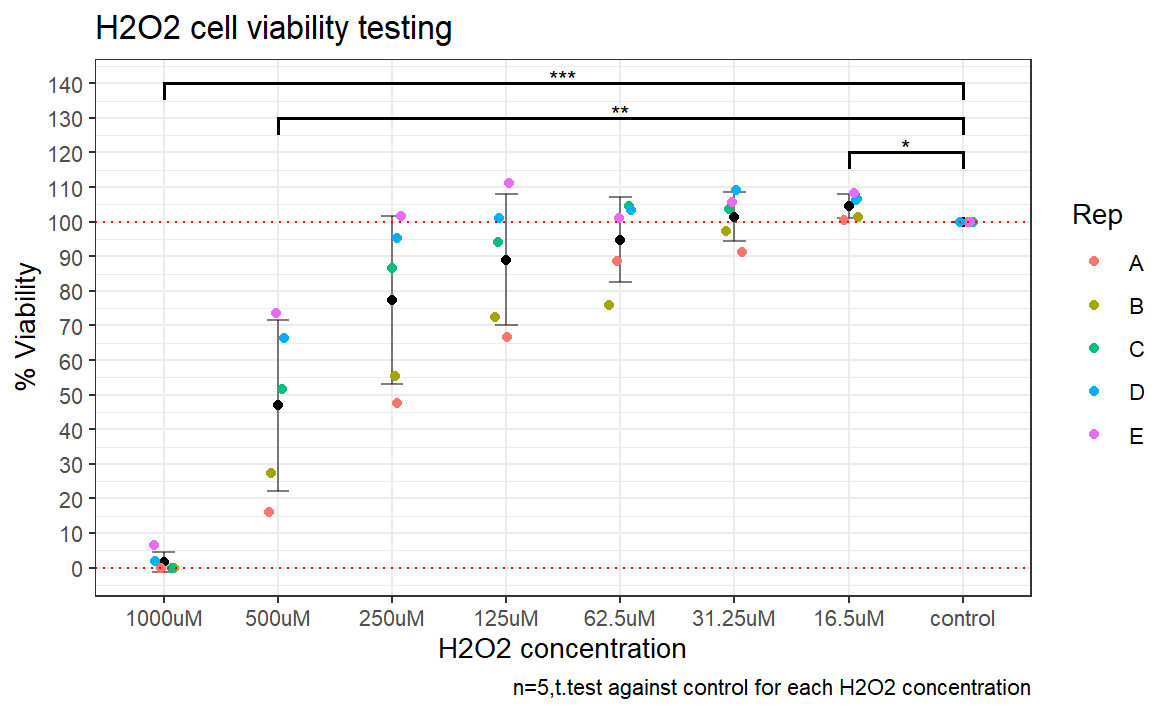


*Supplementary Figure 1: Human NP cell viability after treatment with different concentrations of H_2_O_2_. Viability was estimated using the ratio of treated samples to untreated controls. (n=5, p>0.05: not significant, p<0.05 & p>0.01: *, p <0.01&p>0.001: **, p<0.001: ***)*


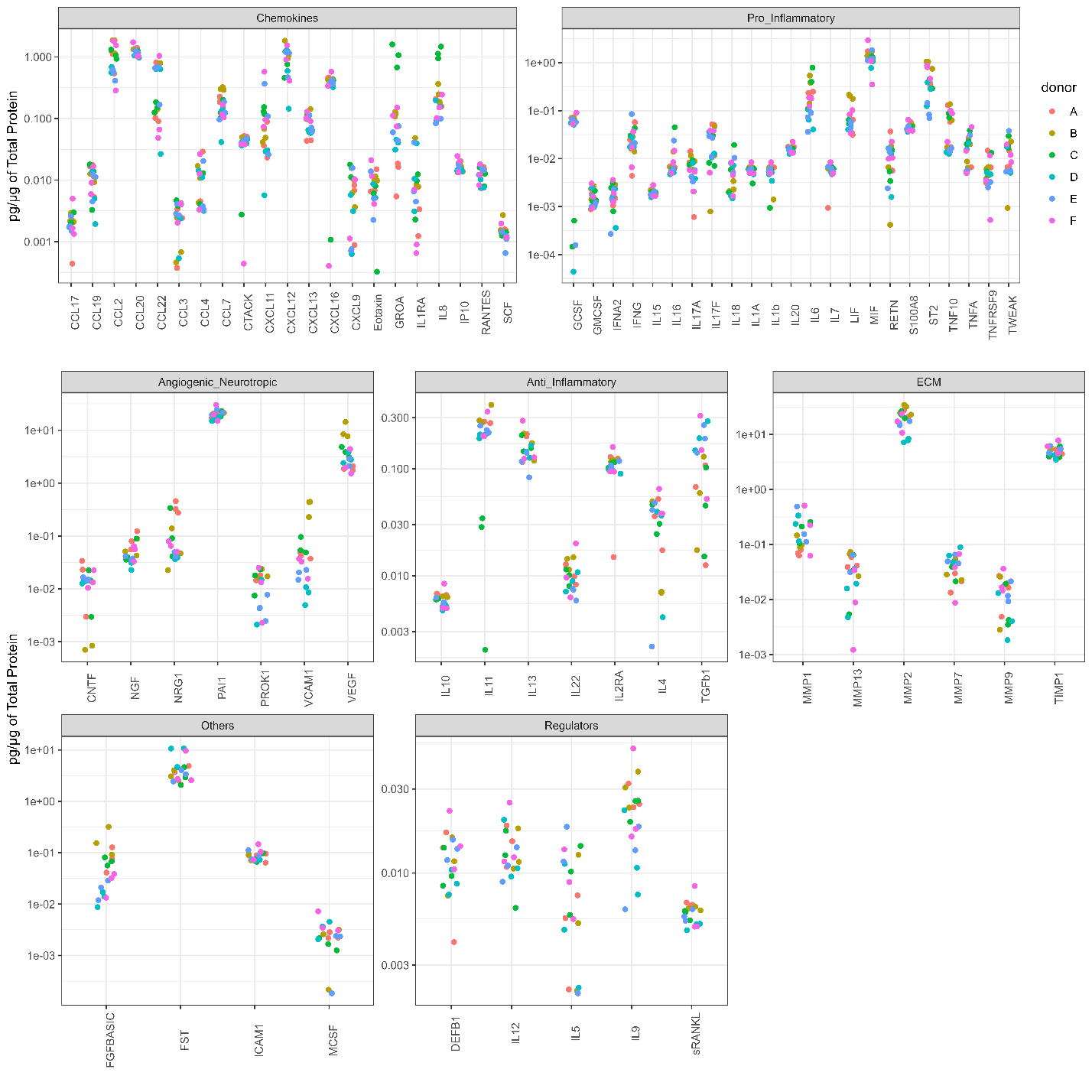


*Supplementary Figure 2: Baseline levels of secreted markers by unstimulated human NP cells after 7 days in monolayer. Protein levels were first normalised to the total protein levels of the corresponding well and then the three replicates of each donor were normalised to their average expression.*


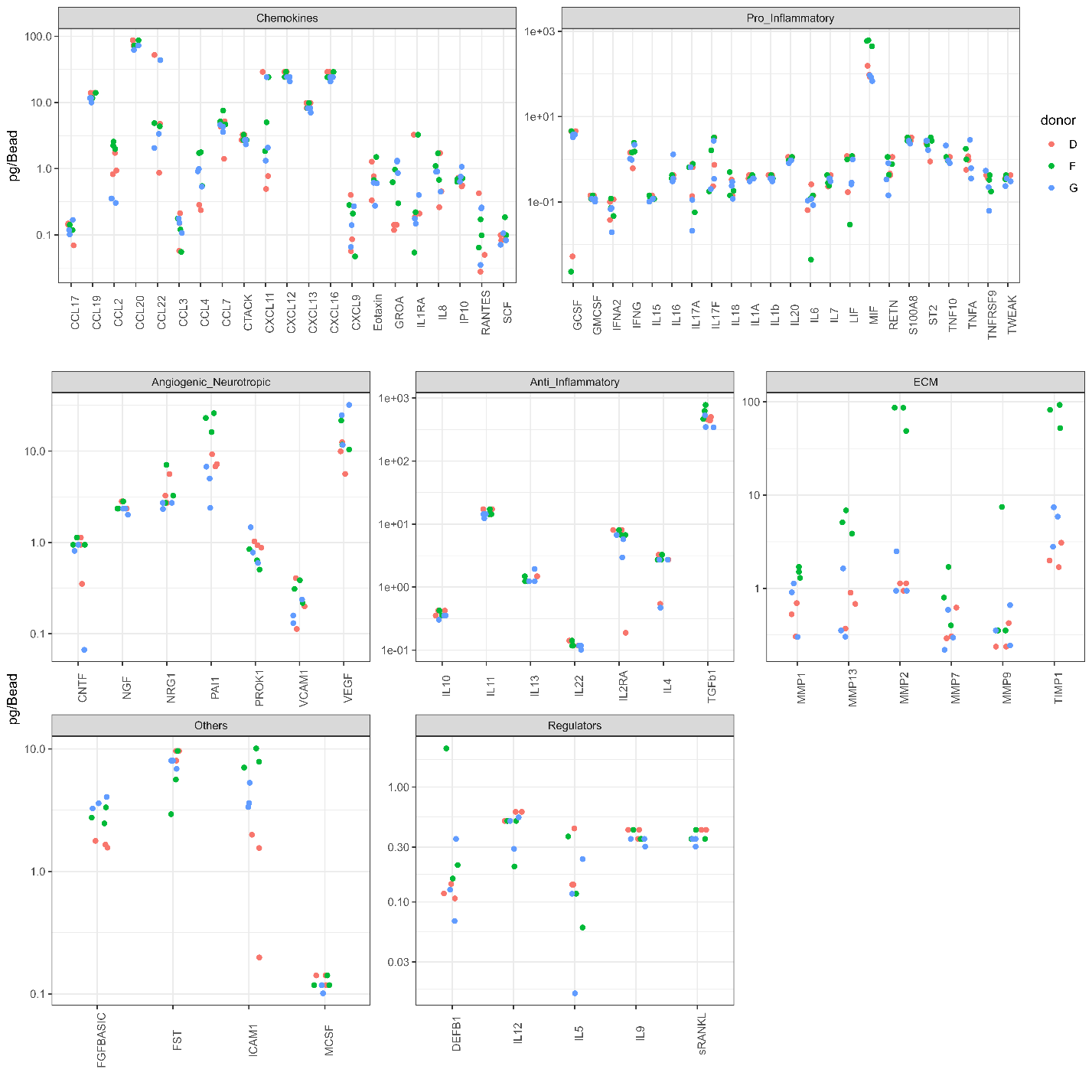


*Supplementary Figure 3: Baseline levels of secreted markers by unstimulated human NP cells after 7 days in alginate. Protein levels were first normalised to the number of beads of the corresponding well and then the three replicates of each donor were normalised to their average expression.*
